# Therapeutic potential of polyphenols in managing thalassemia: A comprehensive review

**DOI:** 10.22038/ajp.2025.26013

**Published:** 2026

**Authors:** Sajjad Jamali, Behzad Jamali, Marjan Golabi, Mehdi Rostami, Zahra Yousefi, Reza Dabbaghipour, Nahid Eskandari, Behrooz Ghezelbash, Sercan Karav, Amirhossein Sahebkar

**Affiliations:** 1 *Drug Applied Research Center, Tabriz University of Medical Sciences, Tabriz, Iran*; 2 *Department of Medical Immunology, School of Medicine, Isfahan University of Medical Sciences, Isfahan-Iran*; 3 *Department of Clinical Biochemistry, Faculty of Medicine, Mashhad University of Medical Sciences, Mashhad, Iran *; 4 *School of Allied Medical Sciences, Shahroud University of Medical Sciences, Shahroud, Iran.*; 5 *Department of Medical Genetics, School of Medicine, Tehran University of Medical Sciences, Tehran, Iran*; 6 *Department of Molecular Biology and Genetics, Canakkale Onsekiz Mart University, Canakkale 17100, Turkey.*; 7 *Applied Biomedical Research Center, Basic Sciences Research Institute, Mashhad University of Medical Sciences, Mashhad, Iran*; 8 *Centre for Research Impact & Outcome, Chitkara College of Pharmacy, Chitkara University, Rajpura 140401, Punjab, India*; 9 *Biotechnology Research Center, Pharmaceutical Technology Institute, Mashhad University of Medical Sciences, Mashhad, Iran*

**Keywords:** β-thalassemiam, Quercetin, Curcumin, Silymarin, Polyphenolic compounds

## Abstract

**Objective:**

This study aims to review the potential effects of polyphenols on iron overload and inflammation in patients with β-thalassemia.

**Materials and Methods:**

A literature search in electronic databases was carried out to identify studies exploring the therapeutic effects of flavonoids in thalassemia.

**Results:**

Patients with thalassemia suffer from excess iron in their bodies. In these patients, splenectomy is usually performed as an effective method to reduce the iron load and the need for blood transfusion. However, since the removal of the spleen in these patients to reduce the excess iron load is faced with serious side effects, it has been suggested to harness iron-chelators. Dietary polyphenols and polyphenol-rich products such as quercetin, curcumin, and silymarin prevent iron overload by reducing the serum levels of iron and ferritin. Polyphenols also reduce inflammation and oxidative stress by reducing tumor necrosis factor-α, C-reactive protein and malondialdehyde, and increasing total antioxidant capacity.

**Conclusion:**

The iron-chelating capacity of polyphenols and flavonoids which may have fewer side effects in patients with thalassemia, has garnered significant attention and holds a promise for therapeutic purposes.

## Introduction

β-thalassemia belongs to a heterogeneous group of genetic disorders characterized by anemia, and it is manifested by reduced or absent production of functional hemoglobin (Needs et al. 2018); singhaTaher et al. (2018). The main symptoms of this disease include ineffective erythropoiesis, chronic hemolytic anemia, and iron overload (Adly and Ismail 2018). More than 300 gene mutations, mainly of the point type, have been found in the binding site and promoter regions of the hemoglobin beta (β)-globin gene on chromosome 11, which leads to defects in the function of this gene and various types of β-thalassemia in people. (Taher et al. 2018). There are three types of β-thalassemia based on the type of β-globin gene mutation. Due to the inheritance of two alleles of two copies of the β-globin gene, if a mutation occurs in one gene, it leads to β-thalassemia minor (β^+^ thalassemia), in which, the production of β chains is severely reduced due to various reasons such as the occurrence of deletion mutations, frameshift mutation, nonsense, and splice-site junction. If the mutation is in two genes, the synthesis of β chains is completely stopped, and it is called transfusion-dependent thalassemia (β0 thalassemia) or Cooley's anemia, which occurs as a result of a mutation in the promoter region (CACCC Box or TATA Box), polyadenylation signal, splicing abnormalities and mutation in the 3ʹ UTR or 5ʹ-UTR regions. The situation between these two types of β-thalassemia is non-transfusion-dependent thalassemia, which involves a slight decrease in β-globin chains (Cao and Galanello 2010; Hamza Bajwa 2022 Jan; Taher et al. 2018).

Approximately 1.5% of the individuals are carriers of β-thalassemia. About 60,000 people with β-thalassemia symptoms are born every year (with an incidence rate of 1 in 100,000 people in the world and 1 in 10,000 people in Europe), with most of them being in developing countries (Galanello and Origa 2010). The reasons for the high prevalence of β-thalassemia include selective pressure caused by *Plasmodium falciparum* malaria, family marriages, the high rate of carriers of this disease, and migration of these people (Galanello and Origa 2010). 

**Figure 1:**
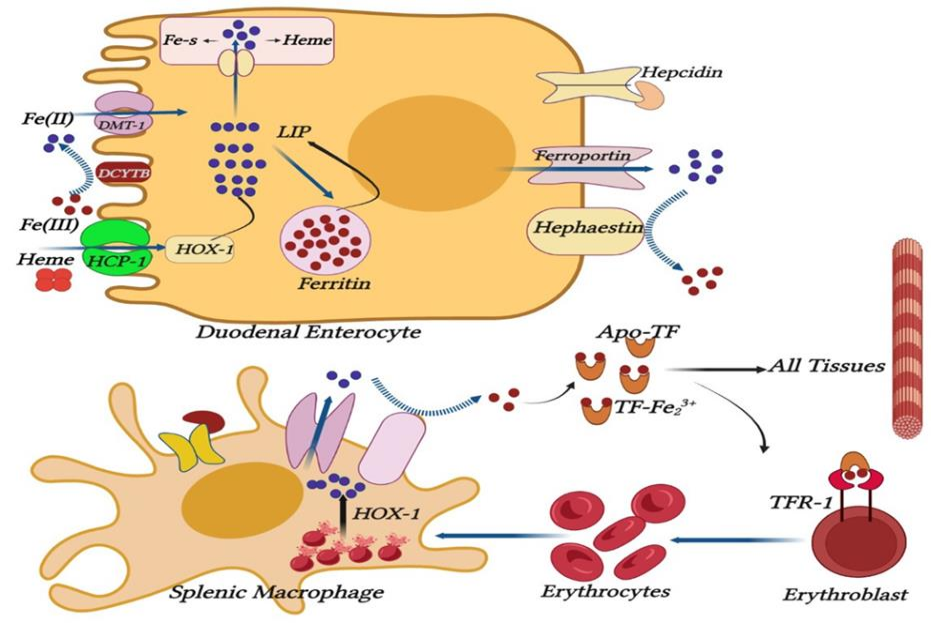
Schematic diagram of the regulation of systemic iron homeostasis. Abbreviations: DCYTB, duodenal cytochrome B; DMT1, divalent metal transporter 1; HCP-1, heme carrier protein-1; Fe(II), ferrous iron (Fe^2+^); Fe(III), ferric iron (Fe^3+^); HMOX1, heme oxygenase 1; Apo TF, iron-free transferrin; TFR1, transferrin receptor 1; LIP, labile iron pool; Fe-S, iron-sulfur.

As shown in the schematic diagram of the regulation of systemic iron homeostasis in Figure 1, the labile iron pool (LIP) consisting of a set of Fe^2+^ is obtained in two ways: 1-Non-heme iron enters enterocyte cytoplasm through heme carrier protein-1 (HCP-1) and is further converted to Fe^2+^ of LIP by heme oxygenase-1. Iron (III) must be reduced to Fe^2+^ to pass through the brush border membrane of duodenal enterocytes to enterocyte cytoplasm, and Fe^2+^ joins LIP through divalent metal transporter (DMT-1). The resulting LIP could be converted to ferritin and *vice versa*, or used in the synthesis of heme and iron-sulfur (Fe-S) clusters, or Fe^2+^ of the LIP enters the portal blood through the basolateral iron exporter, ferroportin (Seyoum et al. 2021). Fe^ 2+^ must be oxidized to Fe^3+^ to bind to iron-free transferrin (Apo TF), mediated by hephaestin ferroxidase. Transferrin transports Fe^3+ ^to the cytosol of all cells through endocytosis by the transferrin receptor-1(TFR-1) and to the erythroblasts of the bone marrow for erythropoiesis (Hentze et al. 2010; Mu et al. 2021; Seyoum et al. 2021). When transferrin is saturated with iron, hepatocytes inhibit iron export through ferroportin degradation by synthesizing and releasing hepcidin (Hentze et al. 2010; Mu et al. 2021). In this disease, iron overload resulting from hemolysis and increased iron absorption from diet along with blood transfusion causes rapid saturation of transferrin and formation of non-transferrin-bound iron (NTBI) or (LIP). These two toxic forms of iron increase the production of free radicals and oxidative damage in the main organs through Fenton's reaction and ultimately cause death (Kalpravidh et al. 2010; Srichairatanakool et al. 2006; Weeraphan et al. 2013). In this disease, hemolytic anemia leads to bone marrow hyperplasia, splenomegaly, hepatomegaly, and severe stimulation of ineffective erythropoiesis. Under these conditions, the synthesis of hepcidin in the liver is suppressed by the erythroid factor erythroferrone, which induces an increase in the concentration of ferroportin, and increases in intestinal iron absorption, and facilitates the release of recycled iron from the reticuloendothelial system. An increase in the levels of serum iron also leads to its progressive deposition in the tissues, and dysfunction of organs, which in acute conditions accelerates the death of the patients (Adly and Ismail 2018; Chaneiam et al. 2013).

The first step in diagnosing any disease after family history is to evaluate its symptoms and clinical manifestations, and later analyze the related laboratory findings. Clinically, patients with β-thalassemia minor are usually asymptomatic but sometimes they have mild anemia (Galanello and Origa 2010). Patients with transfusion-dependent thalassemia are healthy at birth, but after 3 months, when the gamma chain of hemoglobin is replaced by β-chain, symptoms such as jaundice, growth delay, hepato-splenomegaly, endocrine abnormalities, and severe anemia occur, which lead to lifelong blood transfusions (Hamza Bajwa 2022 Jan). In the clinical steps of clinical testing for the diagnosis of thalassemia, performing a complete blood count (CBC) test is a priority. Thalassemia major is characterized by reduced Hb level (< 7 g/dl), mean corpuscular volume (MCV) > 50 < 70 fl which represents the average size of red blood cells, and mean corpuscular Hb (MCH) > 12< 20 pg which shows the average amount of a protein called hemoglobin in each of the red blood cells. Thalassemia intermedia is characterized by Hb level between 7 and 10 g/dl, MCV between 50 and 80 fl, and MCH between 16 and 24 pg. Thalassemia minor is characterized by reduced MCV and MCH (Al-Hakeim et al. 2014). In a study that evaluated iron levels in thalassemia, serum iron, and ferritin levels increased but unsaturated iron binding capacity (UIBC), total iron binding capacity (TIBC), and transferrin saturation percentage decreased, which suggests that these changes rule out iron deficiency anemia (Al-Hakeim et al. 2014). In peripheral blood smear of thalassemia, morphological changes of RBCs such as microcytosis, hypochromia, poikilocytosis, anisocytosis, and nucleated RBCs can be observed. In addition, target cells, tear drop cells, basophilic stippling, Heinz bodies, and a high percentage of reticulocytes can also be seen (Brancaleoni et al. 2016; Cao and Galanello 2010; Hamza Bajwa 2022 Jan; Needs et al. 2022). The hemoglobin of adults includes 3 types of hemoglobin A, A2, and F. The amount of hemoglobin A (HbA) in adults is usually around 95-98%. Hemoglobins A2 and F constitute 2%-3% and less than 2% of adults hemoglobin, respectively (Hamza Bajwa 2022 Jan). In hemoglobin electrophoresis, people with transfusion-dependent thalassemia usually have a high percentage of HbA2 and HbF with the absence or low percentage of HbA. Patients with thalassemia minor generally have a slight increase in HbA2 and a slight decrease in HbA (Hamza Bajwa 2022 Jan). In addition, DNA genetic test analysis with different PCR-based techniques is decisive for confirming mutations in β-globin producing genes and diagnosing thalassemia.

**Figure 2:**
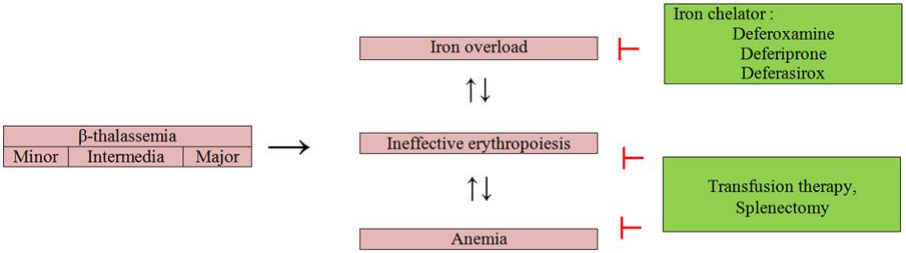
Current treatment strategies in patients with types of β-thalassemia.

Patients with β-thalassemia have different degrees of anemia. In β-thalassemia minor, anemia is mild, but sometimes patients need blood transfusion due to surgery and childbirth (Hamza Bajwa 2022 Jan). In patients with transfusion-dependent thalassemia, chronic hemolytic anemia is severe and they need regular blood transfusions from early childhood. In people with non-transfusion-dependent thalassemia, anemia is often moderate, and occasionally some of them need blood transfusions (Kattamis et al. 2020). Common treatment strategies in β-thalassemia types are shown in Figure 2. In β-thalassemia patients, splenectomy is usually performed to improve growth, quality of life, and hemoglobin level, reduce blood transfusion and iron overload, as an alternative treatment for blood transfusion (Rachmilewitz and Giardina 2011). However, serious side effects such as infection, sepsis, venous thrombosis, pulmonary hypertension, leg ulcer, and stroke have been observed in β-thalassemia patients who underwent splenectomy (Adly and Ismail 2018). Transfusion therapy is a suitable option to deal with complications caused by anemia, hemolysis, and ineffective erythropoiesis, but secondary iron overload is the biggest challenge in treatment with blood transfusion. Currently, to remove excess iron in patients with thalassemia, iron chelators such as deferoxamine (DFO) in the form of subcutaneous or intravenous injection, oral deferiprone (DFP) in the form of tablets or solution, and deferasirox (DFX) in the form of dispersible tablets and coated tablets, are used (Taher et al. 2018). However, the problems in the treatment with chelators have limited their use. Treatment of patients with the most common iron chelator, DFO, may lead to issues such as non-compliance dwith intravenous infusions. Additionally, there is a high incidence of hernias, and some thalassemia patients do not respond adequately to this treatment. The other two iron chelators are oral and are effective only when used in combination with DFO (Balouchi et al. 2014; Poggiali et al. 2012; Thephinlap et al. 2007). Thus, identification of safe and effective natural oral iron chelators with plant origin such as flavonoids as an alternative approach to prevent complications and premature death in thalassemia patients with iron overload seems necessary. Therefore, this review aims to highlight the potential effects of flavonoids in thalassemia.

## Results

### Polyphenols

Polyphenols and in particular flavonoids are secondary metabolites that are widely found in raw plant materials and have numerous health benefits against a wide array of diseases and pathological states (Hasnat et al. 2024; Hosseini et al. 2021; Momtazi et al. 2017; Parsamanesh et al. 2021; Roy et al. 2022; Sahebkar et al. 2016). Due to their strong affinity to bind to iron ions, these substances have multiple biological effects, such as strong antioxidant ability, anti-inflammatory effects, and ability to eliminate free radicals, as well as iron chelation in pathological conditions caused by oxidative stress and iron overload. By strongly binding to iron and forming a complex, flavonoids prevent the increase of free iron and the production of free radicals during the Fenton reaction of iron (Fe^2+^ + H_2_O_2_ → Fe^3+^ + ^⁰^OH + OH^‒^) (Gharagozloo et al. 2013b). In recent studies on flavonoids, these natural substances have a significant role in managing and regulating excess iron load in people who suffer from excess iron by reducing the serum level of iron and ferritin (Sajadi Hezaveh Z 2019, Saeidnia et al., 2022, Reisi et al., 2022). The most common polyphenols in the diet are quercetin, curcumin, and silymarin. Since no toxicity or side effects have been observed with these flavonoids in clinical trials in people (Jiang et al. 2010), these substances could be a suitable option to eliminate iron overload in patients.

### Quercetin

#### Chemistry and structure 

**Figure 3:**
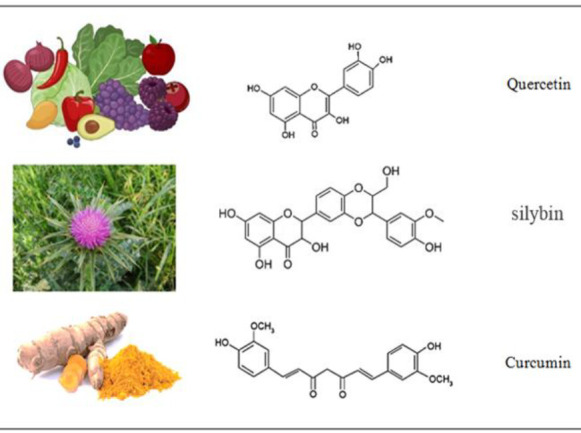
Molecular structures and plant forms of bioflavonoids.

Quercetin is a polyhydroxyflavonoid with the chemical name 2-(3,4-dihydroxyphenyl)-3,5,7-trihydroxychromen-4-one or 3,3′,4′,5,7 pentahydroxyflavone, and three-ring structure. Two rings "A" and "B" are aromatic, but ring "C" is heterocyclic (Derosa et al. 2021; Yin et al. 2021). Benzene rings A and B are connected through the C pyrone ring. Quercetin has a unique structure due to 3-OH and 5-OH groups in A-C rings, 3',4'-dihydroxy groups (catechol part) in the B ring and double bonds (Bardestani et al. 2021). This flavonol is not produced in the human body and is abundantly found in vegetables and fruits such as berries, red grapes, cherries, capers, kale, broccoli, cilantro, dill, apples, and onions (Figure 3). Quercetin is yellow in color, highly soluble in lipids and alcohol, and insoluble in cold water, and it has low solubility in hot water. Dietary quercetin is mainly in the form of glycosides, in which glucose, rhamnose, galactose, and other sugars are conjugated to the 3-hydroxyl position of quercetin through O-glycosidic bonds. As quercetin is lipophilic, it probably crosses enterocytes by passive diffusion. After absorption, the hydrolysis of its glucose group by the β-glycosidase enzyme of intestinal cells or colon bacteria leads to the formation of aglycone which enters the intestinal lumen through the intestinal glucose transporter (SGLT1). Aglycone and its metabolites are transferred to the liver and the resulting metabolic products such as methyl, glucuronide, and sulfate metabolites are distributed into different tissues via the bloodstream (Bardestani et al. 2021; Ezzati et al. 2020; Xiao et al. 2018). Having o-glycosidic double bonds and the hydrophobic nature of quercetin are important advantages for the absorption and passage of quercetin to the target site, i.e. enterocytes.

### Anti-inflammatory and antioxidant effects of quercetin

Quercetin and its metabolites and glycosides have anti-inflammatory and antioxidant activity. After quercetin consumption, its metabolites present in the systemic circulation might act as effective antioxidants and anti-inflammatory agents and this could be considered when defining the overall organic activity of quercetin-rich foods *in vitro* and *in vivo* (Lesjak et al. 2019). Reactive oxygen species (ROS) are highly reactive chemicals consisting of diatomic oxygen (O2), water, and hydrogen peroxide. Some prominent ROS include hydroperoxide (O2H), superoxide (O2^-^), hydroxyl radical (OH), and singlet oxygen. By taking an electron, oxygen becomes a reactive superoxide anion, which the superoxide dismutase (SOD) enzyme converts to hydrogen peroxide. The peroxisomal enzyme catalase (CAT) converts hydrogen peroxide into water and oxygen by reducing glutathione. Glutathione peroxidase (GPx) converts hydrogen peroxide to water by reducing glutathione. This is mainly done by affecting enzyme activity, (ROS), signaling pathways, and inhibition of lipid peroxidation (Xu et al. 2019). Lipid peroxidation is a chain reaction mediated by free radicals that causes increased damage to cell membranes (Tan and Norhaizan 2019). Aggregation of ROS by reducing the activities of antioxidant enzymes, reducing glutathione (GSH), and increasing the lipid peroxidation of the RBC membrane leads to oxidative stress and increased inflammation by activating transcription factors related to inflammation (Darvishi-Khezri et al. 2017; Peng et al. 2021). Quercetin reduces ROS and its effects by donating electrons to ROS and forming more stable and less reactive species (Bardestani et al. 2021). By inhibiting ROS, quercetin increases the expression of endogenous antioxidant enzymes, including CAT, SOD and GPx. It also counteracts the inducing effects of ROS on inflammation by regulating nuclear factor erythroid 2 (Nrf2), AMP-activated protein kinase (AMPK), mitogen-activated protein kinases (MAPK), and NF-KB. Quercetin prevents ROS-induced apoptosis by suppressing the apoptotic proteins P53 and Bax (de Carvalho AT 2020 Aug 26; Xu et al. 2019) (Figure 4). In addition, quercetin inhibits lipid peroxidation by inhibiting lipid peroxyl radicals (ROO^-^) through OH groups in rings A-C and the catechol part in ring B (Metodiewa D 1999). Quercetin acts as an antioxidant and prevents cell inflammation and apoptosis by inflammatory and apoptotic factors (iron overload).

**Figure:**
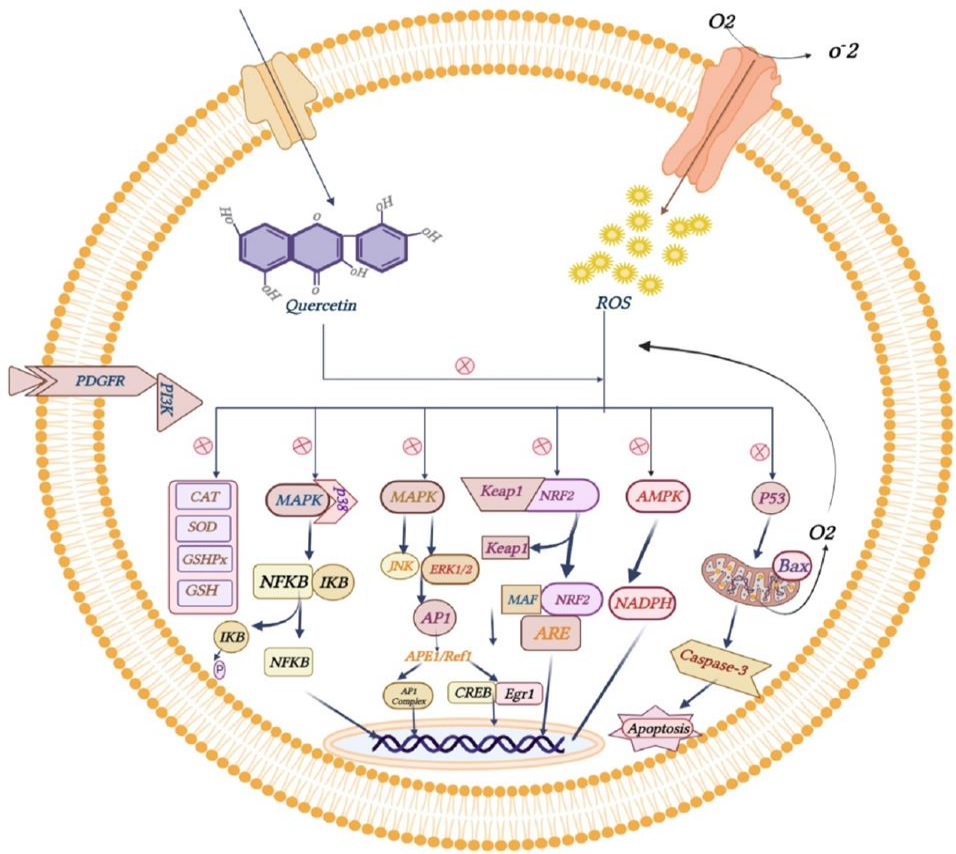


### Quercetin and thalassemia

Two important issues arise when anemia in patients with β-thalassemia is treated with transfusion, including iron overload and oxidative damage caused by high iron, which must be resolved for treatment (Darvishi-Khezri et al. 2017; Hagag AA 2015). Quercetin can reduce hepatic iron levels in patients with β-thalassemia. In addition to reducing iron, it leads to a decrease in ferritin and transferrin saturation and an increase in transferrin (Sajadi Hezaveh Z 2019 -a; Sajadi Hezaveh Z 2019 -b). Quercetin also increases serum non-heme iron levels and promotes the excretion of iron through feces by releasing iron from ferritin. Quercetin has antioxidant activity that inhibits lipid peroxidation and liver protein oxidation caused by iron overload (Sajadi Hezaveh Z 2019 -b).

### Effects of quercetin on iron overload

Iron chelation therapy plays an important role in removing excess iron and maintaining iron homeostasis (El-Sayed EHK 2019). As a potent phytochelator, it can bind to iron ions (Hatcher HC 2009). In the structure of quercetin, there are sites for binding to iron ions, which include the 3-hydroxy-4-ketone group in the C ring, the 5-hydroxy-4-ketone group in the A and C rings, and the catechol part with two hydroxyl groups in the "B" ring (Cheng IF 2000; Guo M 2007; Xiao et al. 2018). Quercetin causes iron chelation by binding to iron ions and forms a quercetin-iron complex. Systemic delivery of absorbed intestinal iron from the enterocyte basement membrane into the circulation is carried out by Fe^2+^ exporter ferroprotein (FPN) and hephaestin ferroxidase, which is responsible for converting Fe^2+^ to Fe^3+^ to bind iron to apotransferrin. By inhibiting the expression of enterocyte basement membrane proteins - FPN and hephaestin, quercetin prevents the transfer of intestinal iron to the blood circulation and its deposition in body organs (Hoque and Sharp 2010; Lesjak et al. 2014). Excess iron-induced ROS causes the separation of Nrf2 from its repressor (Keap1), translocation of Nrf2 to the nucleus, and stimulation of bone morphogenetic protein 6 (BMP6) expression. BMP6-suppressor of mothers against decapentaplegic (SMAD) signaling pathway stimulates hepcidin expression by hepatocytes (Altamura and Galy 2019). By binding to FPN, hepcidin causes its endocytosis and lysosomal proteolysis, and on the other hand, by reducing transferrin receptor 1 (TfR1) and divalent metal transporter 1 (DMT1), it inhibits the entry of excess iron into the blood circulation (Imam et al. 2017; Lesjak et al. 2019). Therefore, quercetin could prevent iron overload by increasing hepcidin.

### Curcumin

#### Chemistry and structure 

Curcumin is a yellow-colored active polyphenolic compound, obtained from the rhizomes of *Curcuma longa* and is known as diferroylmethane (Figure 3). Curcumin has a low molecular weight and comprises about 2-8% of turmeric (Ghaneifar et al. 2020; Tan and Norhaizan 2019; Zia et al. 2021). Curcumin has a hydrophobic nature and is soluble in organic solvents such as dimethyl sulfoxide, methanol, ethanol, and acetone. Curcumin has a high solubility in alkaline conditions and is completely stable in the acidic pH of the stomach (Kim and Clifton 2018; Kotha and Luthria 2019). Several studies have shown that curcumin has strong biochemical and biological activities, including anti-inflammatory, antioxidant, and anticancer activities (Fereydouni et al. 2019; Fu et al. 2021; Ganji et al. 2021; Hamzehzadeh et al. 2018; Panahi et al. 2019; Panahi et al. 2016; Rezaee et al. 2017; Sahebkar and Henrotin 2016; Shafabakhsh et al. 2019). Due to its antioxidant effect, curcumin protects cells against lipid peroxidation, protein carbonylation, and mitochondrial permeability transition (Farkhondeh et al. 2019; Ghaneifar et al. 2020; Kocaadam and Şanlier 2017).

### Anti-inflammatory and antioxidant effects of curcumin

Owing to its anti-inflammatory activity, curcumin is known as one of the natural compounds with the greatest potential in the treatment of various disorders (García-Niño and Pedraza-Chaverrí 2014; Ghaneifar et al. 2020; Kahkhaie et al. 2019; Panahi et al. 2012; Saberi-Karimian et al. 2020). Curcumin shows anti-inflammatory effects by regulating the inflammatory signaling pathways, inhibiting the production of free radicals, and preventing the peroxidation of the biological membrane (Benzer et al. 2018; Campbell and Fleenor 2018; Fernández-Lázaro et al. 2020; Sadeghi et al. 2023; Serafini et al. 2017; Shehzad et al. 2017). In addition, curcumin has phenolic and β-diketone functional groups that help it to be an antioxidant and free radical scavenger (Sathyabhama et al. 2022).

Curcumin has an inhibitory effect on nicotinamide adenine dinucleotide phosphate (NADPH) oxidase and the production of O_2_^.^, OH^.^, H_2_O_2_, NO^.^, ROO^.^, ONOO^-^. It also exhibits its inhibitory effect by preventing the destruction of Nrf2 from the ubiquitin-proteasome pathway and increases the activity of antioxidant enzymes such as CAT, SOD, glutathione-S-transferase (GST), and GPx (Figure 5). Due to these effects of curcumin, the production of ROS and the amount of lipid peroxidation is reduced and oxidative stress is suppressed (Peng et al. 2021; Sathyabhama et al. 2022; Trujillo et al. 2013).

**Figure:**
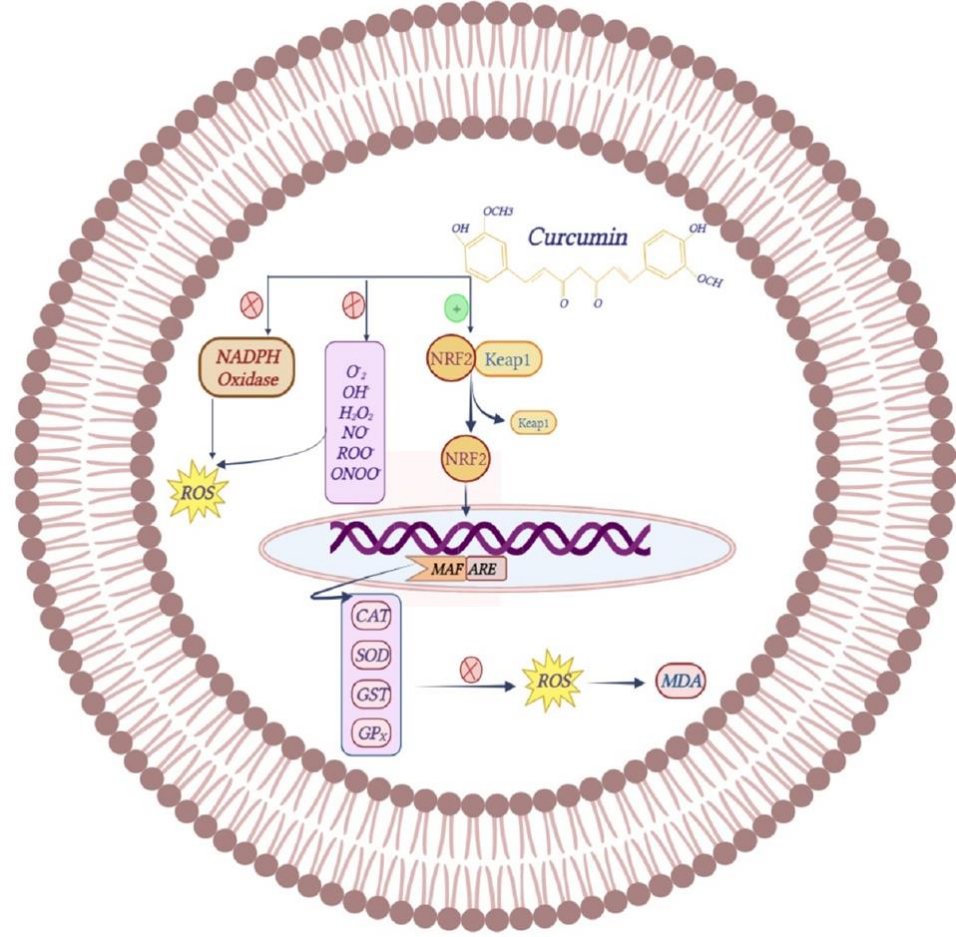


### Anti-thalassemia effects of curcumin

Curcumin has significant iron-chelating effects in patients with iron overload and prevents oxidative damage and organ dysfunction caused by iron overload. Curcumin significantly decreased iron load and liver dysfunction in patients with β-thalassemia by reducing the levels of NTBI, alanine aminotransferase (ALT), and aspartate transferase (AST) (Mohammadi et al. 2018). The use of curcumin in patients with β-thalassemia may lead to a significant decrease in total and direct bilirubin levels as well as serum malondialdehyde (MDA), which indicates a decrease in lipid peroxidation. Curcumin also significantly increased the total antioxidant capacity in these patients (Nasseri et al. 2017). The effects of curcumin in reducing iron overload in patients with β-thalassemia is manifested by a significant decrease in serum iron, ferritin, and transferrin saturation levels (Saeidnia et al. 2022). Overall, curcumin significantly reduces the levels of oxidative stress, hypercoagulability, and iron load in patients with β-thalassemia (Hatairaktham et al. 2021). In addition to reducing MDA and iron bound to non-transferrin, curcumin increases the serum level of GSH in β-thalassemia. Therefore, it reduces oxidative stress and iron overload (Kalpravidh et al. 2010). Consumption of curcumin in heterozygous β-thalassemia mice with iron overload led to increased hemoglobin levels. Moreover, plasma MDA concentrations were significantly decreased following consumption of curcumin in homozygous and heterozygous β-thalassemia mice with extra iron. This finding suggests that curcumin has a protective effect against lipid peroxidation. In addition, a significant decrease in NTBI and cardiac iron accumulation was observed in curcumin-supplemented rats with β-thalassemia with iron overload (Thephinlap et al. 2011). Curcumin consumption in patients with β-thalassemia improves lipid profile and systemic inflammation by reducing triglyceride and high-sensitivity C-reactive protein (CRP) levels (Tamaddoni et al. 2020). In addition, treatment of patients with β-thalassemia with curcumin improved the levels of coagulation factors and iron homeostasis-related proteins, and attenuated oxidative stress indices, which showed the ameliorative role of curcumin against oxidative damage and iron overload (Weeraphan et al. 2013).

### Effect of curcumin on iron overload

Curcumin reduces the amount of iron in the liver and spleen *via* decreasing the levels of hephaestin ferroxidase and increasing DMT1 and TfR1 (Chin et al. 2014). As an excellent metal-chelating ligand, α,β-unsaturated β-diketone moiety of curcumin has a high affinity for binding to the iron target and reducing the amount of iron (Ahmed et al. 2020; Kotha and Luthria 2019). 

### Silymarin

#### Chemistry and structure 

Silymarin is a flavonolignan complex extracted from *Silybum marianum *(L) Gaertneri, commonly known as milk thistle (Figure 3(. Silymarin contains a mixture of several flavonolignans including silybin A, silybin B, isosilybin A, isosilybin B, silychristin, isosilychristin, silydianin and taxifolin flavonoid (Delmas et al. 2020; Hosseinabadi et al. 2019). Silybin A and silybin B are the most abundant and biologically active ingredients of silymarin, which are capable of inhibiting free radicals and lipid peroxidation (Camini and Costa 2020; Hosseinabadi et al. 2019; Jaggi and Singh 2016). Silymarin has been used in the traditional treatment of liver diseases, cirrhosis, and hepatitis, as well as in the protection of liver against toxins (Hosseinabadi et al. 2019).

### Anti-inflammatory and antioxidant effects of silymarin

By suppressing the degradation of κB inhibitor (IκB), silymarin prevents the activation of nuclear factor kappa B (NF-κB) and its transfer to the nucleus to induce the expression of nitric oxide (NO), inducible nitric oxide synthase (iNOS) and cyclooxygenase-2 (COX-2) genes, which are involved in the inflammatory responses. Silymarin also reduces the expression of pro-inflammatory cytokines interleukin-1 beta (IL-1β) and tumor necrosis factor-alpha (TNF-α) (Hou et al. 2010; Lin and Karin 2007; Wang et al. 2002). It also suppresses TNFα-induced ROS production and lipid peroxidation *via* inhibiting the activation of MAPK signaling pathways (Gharagozloo et al. 2013a). Through interactions with the cysteine thiols of Keap1 and activation of protein kinases, silymarin stimulates the phosphorylation of Nrf2 that culminates in the enhancement of antioxidant enzymes such as SOD, CAT, and GPx (Figure 6). Thus, silymarin inhibits both inflammation and oxidative stress (Esmaeil et al. 2017; Surh 2008; Taleb et al. 2018).

**Figure:**
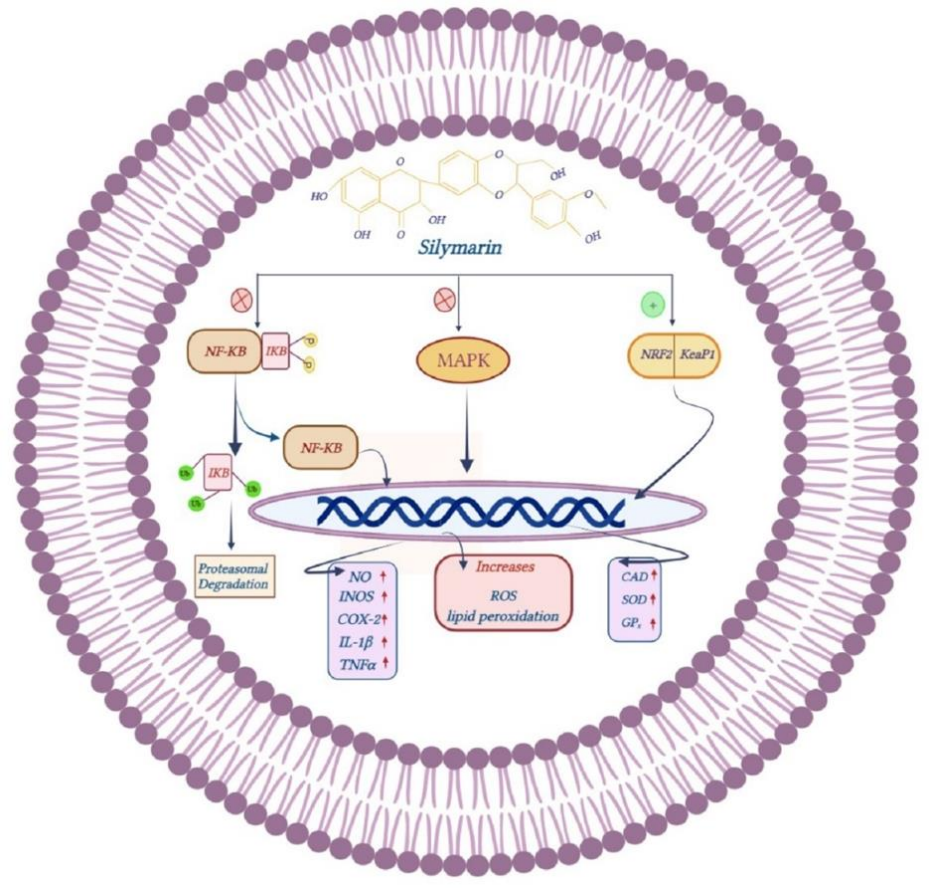


### Anti-thalassemia effects of silymarin

In addition to antioxidant and anti-inflammatory functions, silymarin has chelating effects, which can lead to binding to iron ions and subsequent iron reduction in patients with iron overload. During β-thalassemia-associated oxidative stress, the levels of RBC GSH are decreased while treatment with silymarin could restore that (Alidoost et al. 2006). In patients with β-thalassemia, excessive fat and protein peroxidation causes severe RBC hemolysis (Darvishi-Khezri et al. 2017). In addition, iron overload in these patients causes oxidative stress and a decrease in antioxidant levels (Darvishi-Khezri et al. 2017). Silymarin causes a significant decrease in serum MDA and protein carbonyl and a significant increase in the total antioxidant capacity of serum and plasma GSH in patients with β-thalassemia (Hagag et al. 2013). In addition to increasing GSH in RBCs, silymarin reduces alkaline phosphatase effects and improves liver function (Darvishi-Khezri et al. 2021). Moreover, silymarin reduced serum ferritin levels in patients with β-thalassemia owing to its effects on iron chelation (Darvishi-Khezri et al. 2021). In patients with β-thalassemia, silymarin modulated the immune responses by significantly increasing the anti-inflammatory cytokines IL-23, transforming growth factor beta (TGF-β), and decreasing the inflammatory cytokine IL-10 (Balouchi et al. 2014). In the same patients, the use of oral deferasirox with silymarin led to significantly decreased serum levels of ferritin compared to oral deferasirox and placebo (Hagag et al. 2013). Also, regular consumption of oral DFP and silymarin showed a significant decrease in serum levels of ferritin and iron compared to oral DFP and placebo (Hagag AA 2015). Silymarin was reported to improve the inflammatory status of patients with β-thalassemia by increasing the levels of CRP, IL-6, and IL-10 (Moayedi et al. 2013). In these individuals, silymarin treatment led to a significant decrease in serum iron and ferritin levels as well as a significant increase in TIBC levels. A significant decrease was also observed in the liver iron concentration during treatment with this flavonoid. This data revealed the potential effects of silymarin in improving the status of iron load in patients with β-thalassemia (Ezzati et al. 2020; Hagag AA 2015; Reisi et al. 2022). In addition, due to its anti-inflammatory activity, silymarin plays an important role in the recovery of β-thalassemia patients by reducing inflammatory factors such as TNF-α and neopterin, and increasing anti-inflammatory cytokines interferon-gamma (IFN-γ) and IL-4 (Gharagozloo et al. 2013b). In this hematological disorder, treatment with silymarin significantly decreased the serum levels of ferritin, iron content, TIBC, hepcidin, and soluble transferrin receptor. Additionally, significant improvements in liver function tests were observed with silymarin compared to placebo (Ezzati et al. 2020). These results suggest that silymarin can serve as a natural and safe iron-chelating agent for the treatment of patients with various iron overload disorders such as β-thalassemia.

## Discussion

Flavonoids are a class of phenolic compounds that have revealed biological effects including antioxidant, anti-inflammatory, and free radical-removing activities. Quercetin, curcumin, and silymarin are common dietary polyphenols and polyphenol-rich natural products. Quercetin can reduce ROS production, increase the expression of endogenous antioxidant enzymes, and prevent ROS-induced apoptosis by suppressing P53 proto-oncogene and Bax proteins. In addition, the anti-inflammatory properties of curcumin suggest that this compound is an effective natural product for the treatment of various diseases. It has phenolic and diketone functional groups that aid in its antioxidant and free radical-scavenging abilities. Curcumin also increases the activity of antioxidant enzymes like CAT, SOD, GST, and GPx, while inhibiting NADPH oxidase and the production of OH, NO, ROO, and ONOO species. Silymarin also inhibits the expression of pro-inflammatory cytokines such as IL-1β and TNF-α. Furthermore, by preventing the activation of MAPK signaling pathways, silymarin reduces the production of ROS and lipid peroxidation that are caused by TNF-α. Silymarin tends to increase antioxidant enzymes like SOD, CAT, and GPx by phosphorylating Nrf2 and interacting with the cysteine thiols of Keap1 and activating protein kinases. Therefore, it can prevent oxidative stress. However, more investigations are required to confirm the anti-inflammatory effects of these substances. 

Besides antioxidant and anti-inflammatory functions, these compounds have shown a potential role in iron chelation. The 3-hydroxy-4-ketone group in the C ring, the 5-hydroxy-4-ketone group in the A and C rings, and the catechol component with two hydroxyl groups in the "B" ring are the sites in the structure of quercetin that can bind to iron ions. By forming a quercetin-iron complex with iron ions, quercetin induces iron chelation. The Fe^2+^ exporter ferroprotein (FPN) and hephaestin ferroxidase, which are in charge of converting Fe^2+^ to Fe^3+^ to bind iron to apo-transferrin, are responsible for the systemic delivery of absorbed intestinal iron from the enterocyte basement membrane into the circulation. Quercetin also decreased the transfer of intestinal iron to the blood circulation and its deposit in red blood cells by inhibiting the expression of enterocyte basement membrane proteins FPN and hephaestin. By increasing DMT1 and TfR1 and decreasing hephaestin ferroxidase, curcumin decreased the amount of iron stored in the liver and spleen. An excellent metal-chelating ligand with a high affinity for binding to the iron target and lowering the amount of iron is the unsaturated diketone moiety of curcumin. To treat iron overload, silymarin is also a natural and secure iron-chelating agent. 

Overall, studies have shown that these natural treatments, which have fewer side effects than conventional iron chelators and splenectomy, can be used as safe and effective natural products for the better management of β-thalassemia disorder. However, more clinical studies are needed to support this treatment strategy.
